# Novel Clue to Locate Conduction Gaps in the Pulmonary Vein Isolation Ablation Line

**DOI:** 10.3389/fcvm.2021.622483

**Published:** 2021-07-12

**Authors:** Hai-yang Xie, Xiao-gang Guo, Jian-du Yang, Yan-qiao Chen, Zhong-jing Cao, Qi Sun, Jian Ma

**Affiliations:** State Key Laboratory of Cardiovascular Disease, Arrhythmia Center, Fuwai Hospital, National Center for Cardiovascular Diseases, Chinese Academy of Medical Sciences and Peking Union Medical College, Beijing, China

**Keywords:** atrial fibrillation, pulmonary vein ablation/isolation, catheter ablation–atrial fibrillation, arrhythima, conduction gap

## Abstract

**Background:** Several methods have been reported for locating the conduction gap (CG) in the pulmonary vein isolation (PVI) ablation line. However, the value of the interval between far-field atrial potential (FFP) and pulmonary vein potential (PVP) remains unknown.

**Methods:** Consecutive patients with a CG during observation on the table after PVI were included. The PVP, FFP, and the CG location were evaluated to develop a novel algorithm to identify the CG location in the left superior pulmonary vein. The performance of this novel algorithm was prospectively tested in a validation cohort of consecutive patients undergoing repeat PVI ablation.

**Results:** A total of 116 patients with atrial fibrillation (AF) were recruited, 56 of whom formed the validation cohort. The interval between FFP and PVP of the left superior pulmonary vein was associated with the CG location, and an interval <5 ms predicted the presence of CG in the upper portion of the ostium with a sensitivity of 92.9% and a specificity of 96.9%. In the prospective evaluation, the interval was able to correctly predict the site of CG in 89.6% of cases.

**Conclusions:** The interval between FFP and PVP is a novel and accurate index that can be used to predict the CG location in the left superior pulmonary vein. An far-field atrial potential and pulmonary vein potential (FFP–PVP) interval value of ≥5 ms could be used to exclude a CG in the upper portion of the ostium in the majority of patients undergoing AF ablation.

## Background

Pulmonary vein isolation (PVI) is the cornerstone of catheter ablation in many patients with atrial fibrillation (AF) (1). Pulmonary veins (PVs) are an important source of ectopic beats, initiating frequent paroxysms of AF. Ectopic beats are found frequently, particularly in the left superior pulmonary veins (LSPVs) (47.7%) (2). However, non-isolation after initial PVI during observation on the table or in patients after discharge from the hospital is not rare (3). Reisolation of the recovered PVs has demonstrated improvement in eliminating AF and clinical outcomes (4, 5). Therefore, touch-up is frequently required to eliminate conduction gaps (CGs) (6). During a repeat PVI procedure, identifying CGs is sometimes critical and difficult. Predicting the CG location before ablation can significantly reduce atrial and PV lesions and decrease operation time.

The primary goal of this study was to systematically evaluate the role of the interval between the far-field atrial potential and pulmonary vein potential (FFP–PVP interval) in identifying CGs and to develop a novel algorithm based on these findings.

## Methods

This study was conducted in two phases: (1) a development and hypothesis-generating phase whereby patients' electrophysiology study and Carto 3 systems (Carto, Biosense Webster, Diamond Bar, CA, USA) data were systemically recorded and analyzed during isolation of the LSPV, and then used to create a precise algorithm to predict the CG location; and (2) a prospective validation of this new algorithm. The study was approved by the institutional review board of Fuwai Hospital.

### Patient Selection

Consecutive patients with a CG during observation on the table after PVI were included to create the new algorithm. Patients with LA-LSPV reconnection during repeat PVI were recruited to verify the results. Exclusion criteria were structural heart disease, left atrial diameter ≥50 mm, long-standing (≥1 year) persistent AF, AF that occurred on the table, and a common ostium of left/right PVs. Informed consent was obtained before the procedure.

### Electrophysiology Study and Catheter Ablation

Data were recorded simultaneously by a digital multichannel system (LabSystem PRO, Bard Electrophysiology, Lowell, MA, USA). Bipolar signals were filtered at 30–500 Hz, and unipolar signals were filtered at 0.05–500 Hz. Electroanatomic mapping was performed using Carto 3 systems. A 3.5-mm-tip catheter (ThermoCool SmartTouch, Biosense Webster Inc.) was used for three-dimensional electroanatomic mapping and ablation. The PV ostium was identified by selective venography and focal potential, and tagged on a three-dimensional electroanatomic map. A Lasso catheter (Biosense-Webster, Inc.) was placed at the PV ostium to observe the change in PVP and confirm PVI during radiofrequency (RF) ablation.

The PVI technique has been previously described in detail (1). In all procedures performed by an experienced operator, irrigated RF ablation using a point-by-point technique was initially performed to create right-sided continuous circular lesions around PVs and subsequently on the left side. For the left side, ablation started at the inferior aspect and continued posteriorly, followed by the superior and anterior portions. Radiofrequency ablation was performed in the posterior wall ~1 cm and in the anterior wall ~5 mm from the PV ostium (7, 8). Radiofrequency energy was delivered in a power control mode and titrated from 20 to 35 W, 43°C maximum temperature with an irrigation rate of 17–25 ml/min. The target duration of RF delivery ranged between 30 and 60 s but was terminated in advance if local electrogram attenuation was >70% or double potentials were noted (9). The general consensus is that complete electrical PV isolation is the optimal endpoint when ablation is performed at the PV-LA junction. Once the LSPV was isolated, the PVP and the location of that ablation site were specifically marked for analysis.

For the validation group, the reconstruction of three-dimensional electroanatomic mapping in LA was achieved first, and then the previous electric reconduction of isolated PVs was assessed. Subsequently, PVP activation of the LSPV was recorded, and the CG location was mapped according to our findings. Finally, ablation was performed at which the earliest activation was detected at the Lasso catheter.

### Definitions

**Figure 1:**
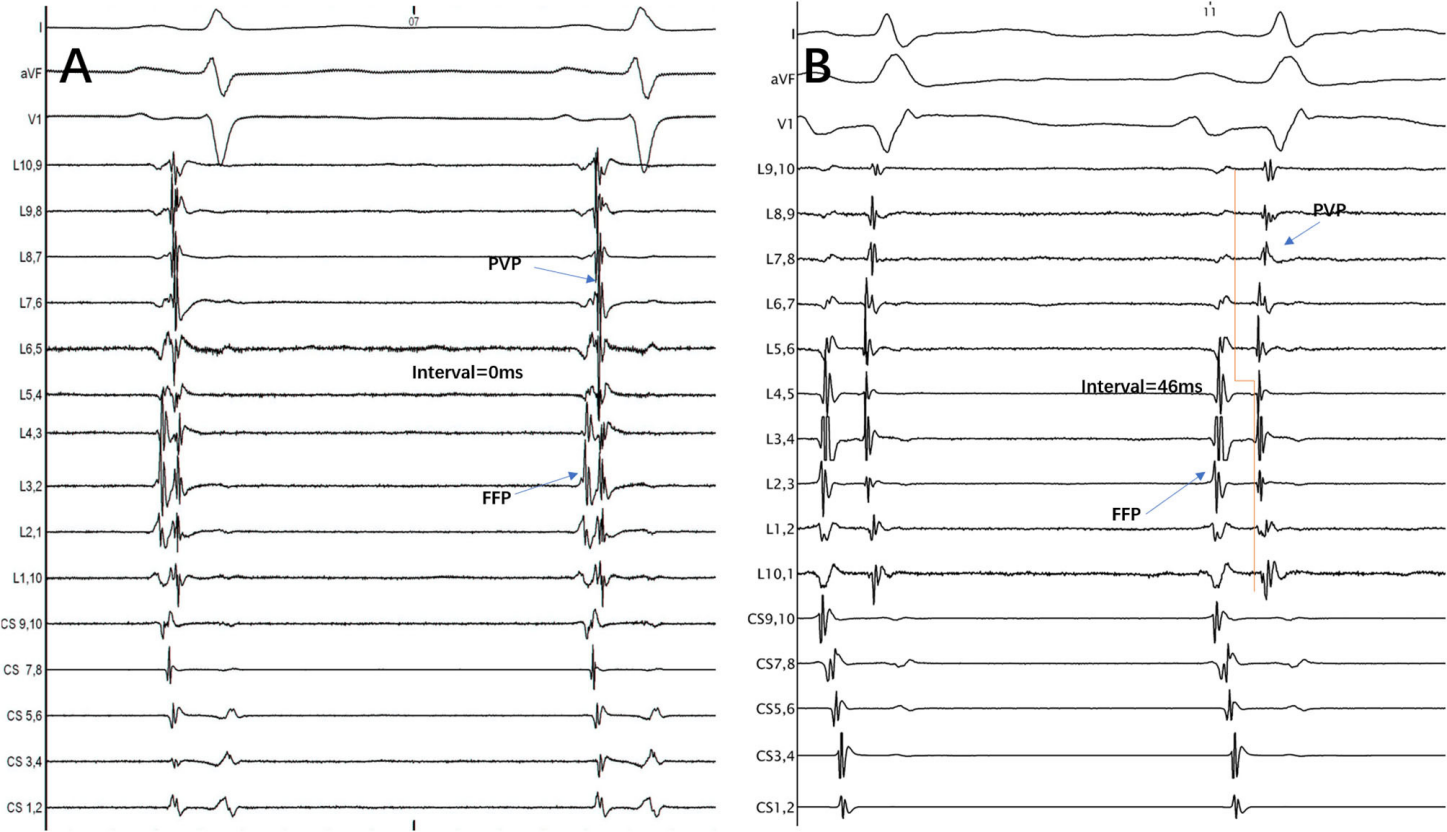
The interval between FFP and PVP at the Lasso catheter was measured from the FFP offset to the earliest PVP onset. **(A)** FFP and PVP overlapped, so the interval between them was defined as the value of 0 ms. **(B)** FFP and PVP were separated, so the interval between them was 46 ms. FFP, far-field atrial potential; PVP, pulmonary vein potential; CS, coronary sinus; L1.2, Lasso catheter distal electrode; L9.10, Lasso catheter proximal electrode.

The FFP–PVP interval was defined as the time from the FFP offset to the earliest PVP onset at the Lasso catheter (Figure 1). If those two potentials overlapped and their offset or onset could not be recognized, the interval was then defined as the value of 0 ms.

According to the anatomical structure, the ostium of the LSPV was divided into two sections: upper and lower portions. In detail, the upper portion was defined as the top half of the ostium while the lower portion was the remaining section. Otherwise, from 10 to 2 o'clock of the ostium was defined as the PV roof.

### Statistical Analysis

Continuous parameters are presented as the mean ±*SD* and were assessed using Student's *t*-test or the Mann–Whitney test. Categorical variables are presented as counts (percentage) and were analyzed using the chi-square test or Fisher's exact test. Sensitivity and specificity for each variable were determined using the receiver operator characteristics (ROC) curve. All clinically relevant variables were included in the multivariable logistic regression to identify the variables that were independently associated with the presence of a second loop. A value of *P* < 0.05 was considered significant. SPSS, version 19.0 (SPSS, Inc., Chicago, IL, USA) was used for statistical analysis.

## Results

### Patient Characteristics

The cohort consisted of 112 patients [96/112 male (85.7%)]. None had long-standing (≥1 year) persistent AF. Patients' characteristics are shown in Table 1.

**Table 1:** Baseline characteristics of patients in the development and prospective cohorts.

	**Development cohort**							**Prospective cohort**			
	**All**	**Upper portion**	**Lower Portion**	**Univariable *P*-value**	**Multivariable *P*-value**	**HR (95% CI)**	**All**	**Upper portion**	**Lower portion**	**Both portion**
Patients, *n* (%)	60	32 (53)	28 (47)	NA			56	28 (50)	20 (36)	8 (14)
Male, *n* (%)	47 (78)	25 (78)	22 (79)	0.967	0.46		49 (88)	24 (86)	17 (85)	8 (100)
Age, years	56.3 ± 8.4	57.1 ± 7.8	55.3 ± 9.0	0.419	0.63		54.1 ± 9.7	56.2 ± 10.1	53.2 ± 9.3	49.1 ± 7.7
LAD, mm	37.1 ± 6.0	36.2 ± 4.7	36.5 ± 4.7	0.892	0.16		37.0 ± 3.5	37.7 ± 3.7	37.6 ± 3.3	37.6 ± 3.1
CHA2DS2-VASc	1.6 ± 1.7	1.9 ± 1.8	1.2 ± 1.5	0.095	0.83		1.3 ± 1.5	1.3 ± 1.4	1.7 ± 1.7	0.3 ± 0.7
FFP–PVP interval	15.1 ± 21.9	0.6 ± 2.6	31.6 ± 22.5	<0.005	0.006	1.55 (1.13–2.13)				

*LAD, left atrial diameter; FFP, far-field atrial potential; PVP, pulmonary vein potential*.

### Development of the Novel Algorithm

**Figure 2:**
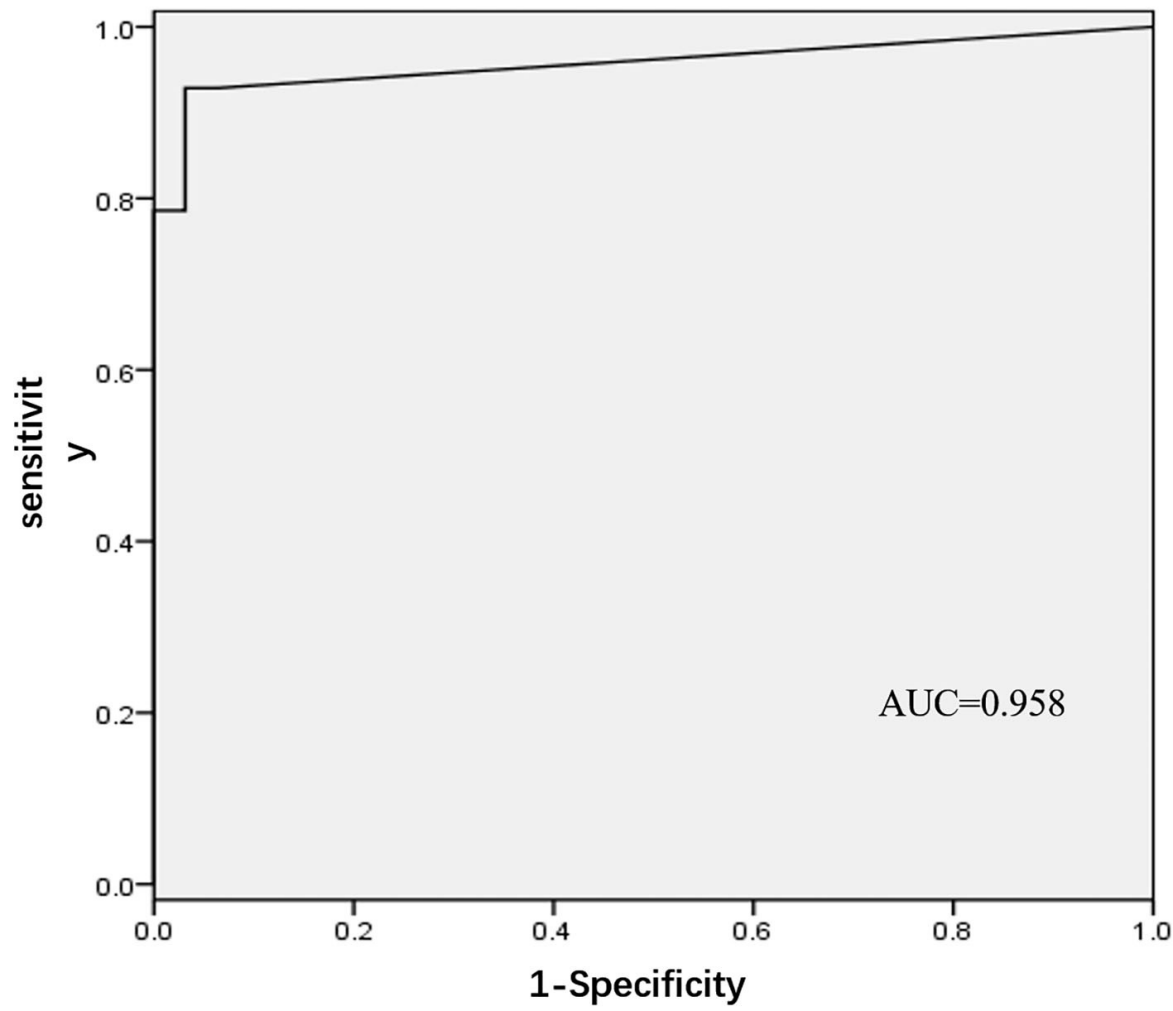
Receiver operating characteristic (ROC) analysis for different intervals between far-field atrial potential and pulmonary vein potential.

In the development phase, 60 patients with AF who underwent PVI ablation in Fuwai Hospital from July 2015 to July 2017 were included in the development cohort. CGs were identified in 32 (53.3%) patients in the upper portion of the ostium, and 28 (46.7%) were identified in the lower portion. In the upper portion, most of the CGs (30/32, 93.75%) were located at the roof. Within the lower portion, all the CGs were located at the junction of PV. There were no significant differences in age, sex, left atrial diameter, or CHA2DS2-VASc between the two groups (Table 1). In the logistic regression analysis, FFP–PVP interval was identified as the only factor associated with the CG location (OR = 1.55, 95% CI 1.13–2.13, *P* = 0.006), which indicated that the interval in the lower portion of the ostium was greater than that in the upper portion. Receiver operator characteristics analysis of the FFP–PVP interval is shown in Figure 2. The AUC was 0.958, and an interval <5 ms predicted that there was a CG in the upper portion of the ostium with a sensitivity of 92.9% and a specificity of 96.9%. Based on the above findings, a preliminary algorithm was developed. Once nonisolation occurs after circumferential PV ablation, the FFP–PVP interval should be measured. If the interval is <5 ms, the CG is likely to be located in the upper portion; otherwise, it is located in the lower portion.

### Validation of Novel Algorithm Incorporating Interval Index

**Figure 3:**
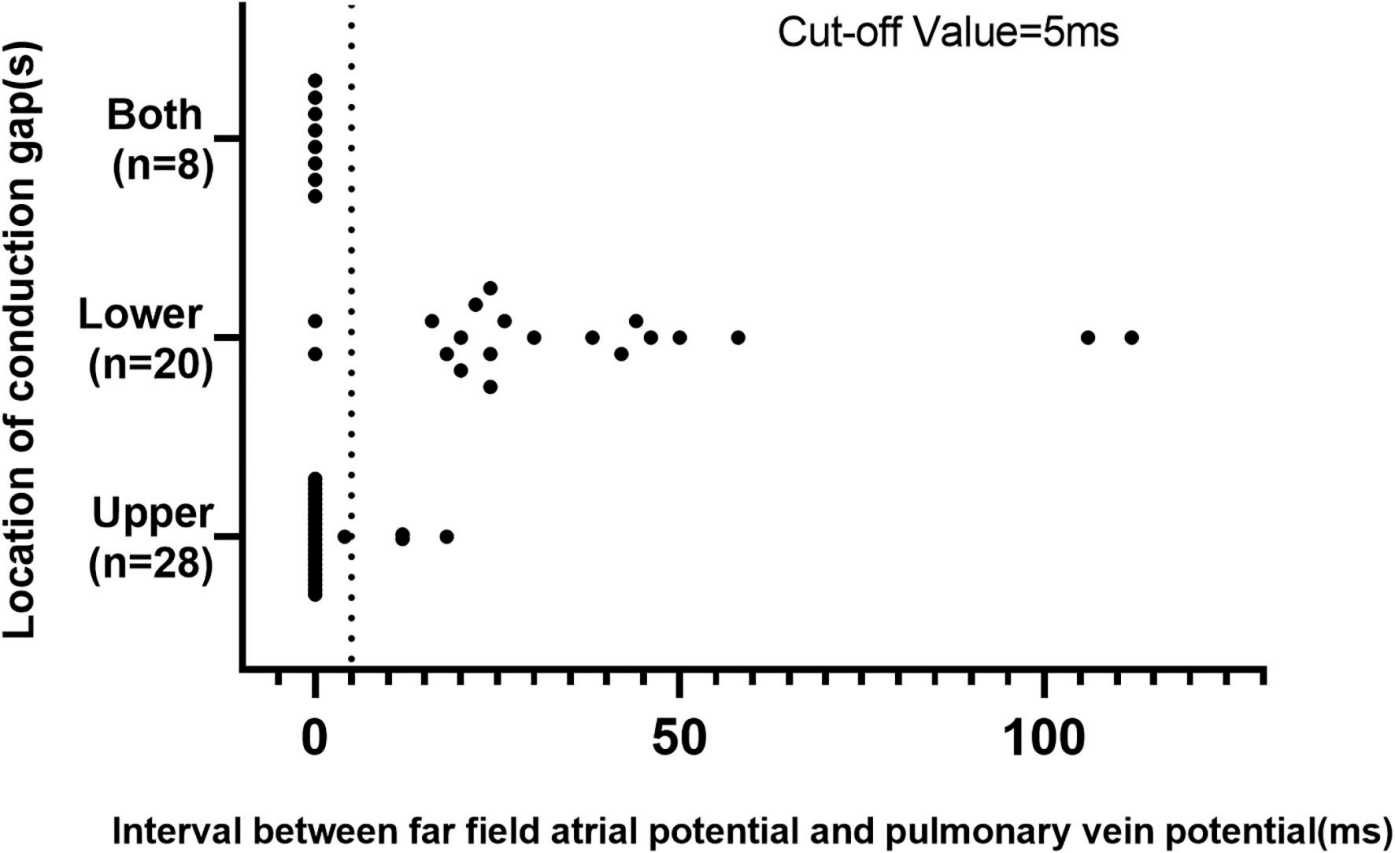
Scatter plot of the cutoff value of the interval between far-field atrial potential and pulmonary vein potential in the validation cohort.

**Figure 4:**
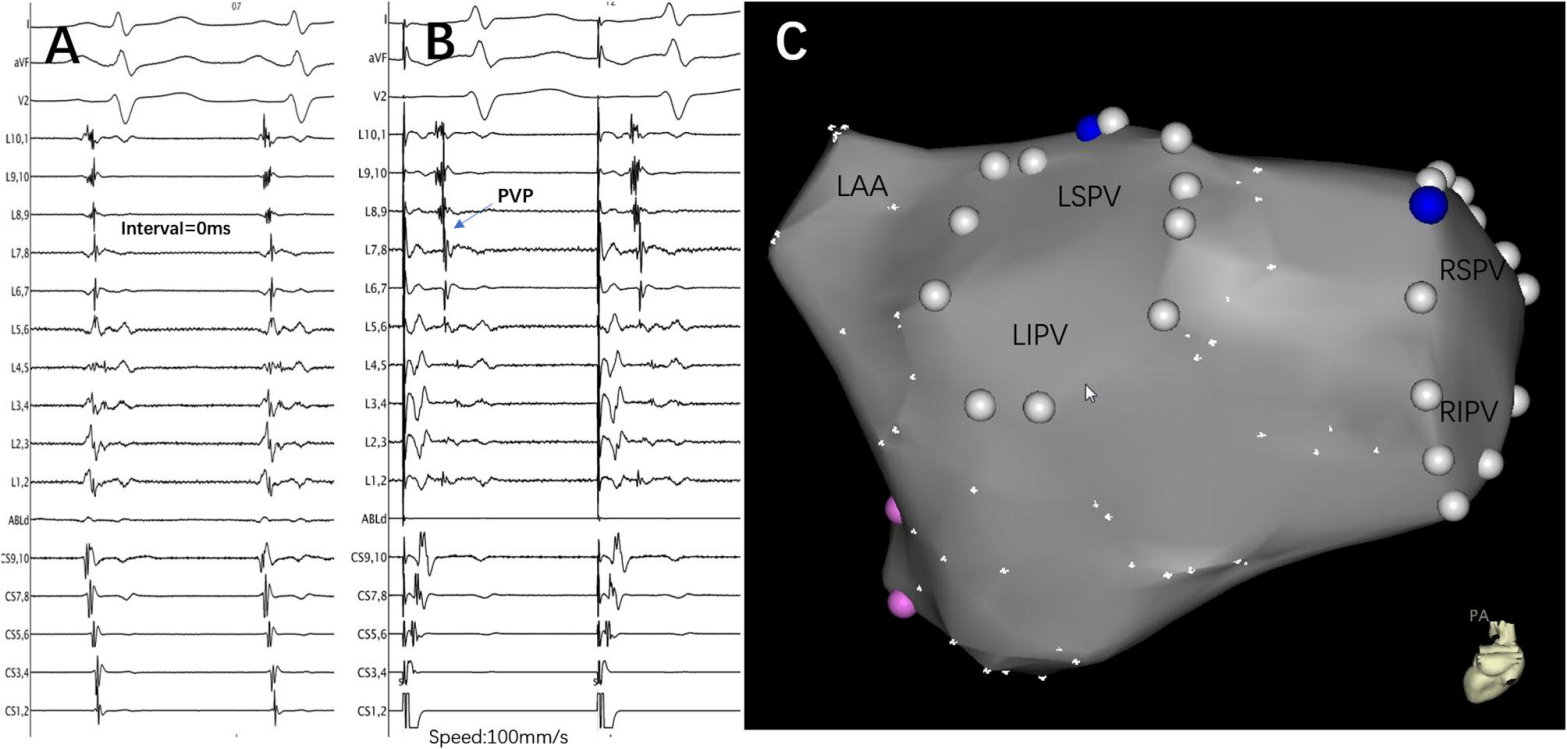
An example of a conduction gap that was correctly predicted by the interval in a re-do patient. **(A)** Lasso was placed at the ostium of the LSPV, and the PV and left atrium were reconnected. The interval between FFP and PVP was 0 ms. **(B)** Pacing at the distal CS catheter to distinguish and confirm the existence of PVP. **(C)** The successful ablation target was only located at the roof of the LSPV. The blue point represents the ablation target, the white point represents the pulmonary vein ostium, and the pink point represents the mitral valve. LSPV, left superior pulmonary vein; LIPV, left inferior pulmonary vein; RSPV, right superior pulmonary vein; RIPV, right inferior pulmonary vein; PV, pulmonary vein; LAA, left atrial appendage; CS, coronary sinus; FFP, far-field atrial potential; PVP, pulmonary vein potential; L1.2, Lasso catheter distal electrode; L9.10, Lasso catheter proximal electrode.

From September 2017 to September 2019, 56 patients with recurrent AF and reconnected LA-LSPV were recruited in the validation cohort. Individual CG was found in the lower portion of the ostium in 20 patients and the upper portion in 28 patients. Eight patients had CGs in both portions. The scatter plot of cutoff values of the FFP–PVP interval in the validation cohort is shown in Figure 3. In 18 of 20 (90.0%) patients with the CG located in the lower portion of the ostium, the FFP–PVP interval was >5 ms, whereas in 25 of 28 (89.3%) patients with the CG located in the lower portion, the FFP–PVP interval was shorter than 5 ms. As such, this cutoff value was able to correctly predict the site of CG (upper portion versus lower portion of the ostium) in 89.6% (43 of 48) of cases, with a sensitivity of 92.6% and a specificity of 85.7%. An example of a CG that was correctly predicted by the interval in a re-do patient is shown in Figure 4.

It is also interesting to note that in patients with CG located in both portions, the FFP–PVP intervals were all shorter than 5 ms. After closing the CG at the upper portion, a new CG was identified with a delay in the timing (22.3 ± 13.0 ms); all were eventually eliminated in the lower portion.

## Discussion

### Major Findings

The major finding of this study is that the interval between FFP and PVP of the LSPV provided a more accurate location of the CG on the PVI ablation line. The key findings in this series are as follows: (1) The upper portion of the LSPV is the dominant conduction area for the LSPV during sinus rhythm. (2) An FFP–PVP interval <5 ms predicted the presence of CG in the upper portion of the ostium with a sensitivity of 92.9% and a specificity of 96.9%.

### Possible Anatomic Considerations

**Figure 5:**
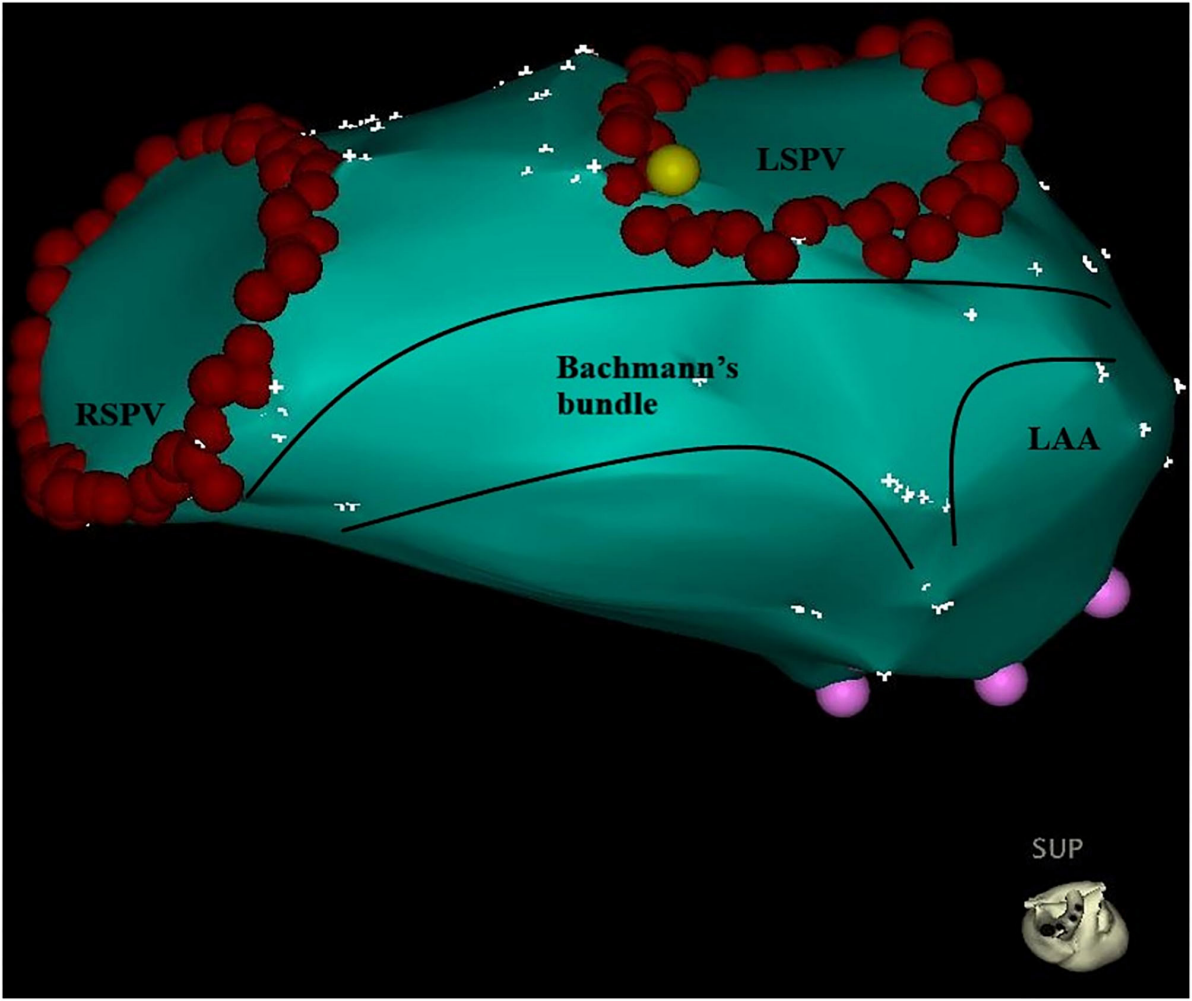
Bachmann's bundle branches to the left atrium and passes around the neck of the left atrial appendage, reuniting to continue into the musculature of the lateral and posteroinferior atrial walls. The red point represents the pulmonary vein ostium. LSPV, left superior pulmonary vein; RSPV, right superior pulmonary vein; LAA, left atrial appendage.

Our findings might be explained by anatomical structure and interatrial conduction. Usually, Bachmann's bundle (Figure 5), known as the interatrial band, is composed of a nearly parallel alignment of myocardial strands, which accounts for its role as the prevalent interatrial conduction pathway for propagation of the sinus impulse to the anterior left atrial wall. To the right, Bachmann's bundle connects the right atrial appendage (AA), and the superior arm can extend toward the sinus node. To the left, Bachmann's bundle branches to pass around the neck of the left AA, reuniting to continue into the musculature of the lateral and posteroinferior atrial walls (10). Therefore, as for the LSPV, the earliest breakthrough usually occurs at the roof and then the impulse travels down anteriorly and posteriorly to the inferior portion. The left AA is activated simultaneously when LSPV is activated from the upper portion. Therefore, PVP and FFP in general overlap without any intervention. Once the upper portion was blocked, especially the roof, LSPV could be activated only from the lower portion where a CG existed. As a result, the activation pathway of the LSPV was prolonged, and PVP separated from FFP. Conversely, if the upper portion was not blocked, the activation pathway remained unchanged, and the FFP–PVP interval would not change. This agrees with the results of the present study on the prediction of CG in the upper portion of the ostium, using the cutoff value of an FFP–PVP interval <5 ms.

### Strategy for Locating CGs

**Figure 6:**
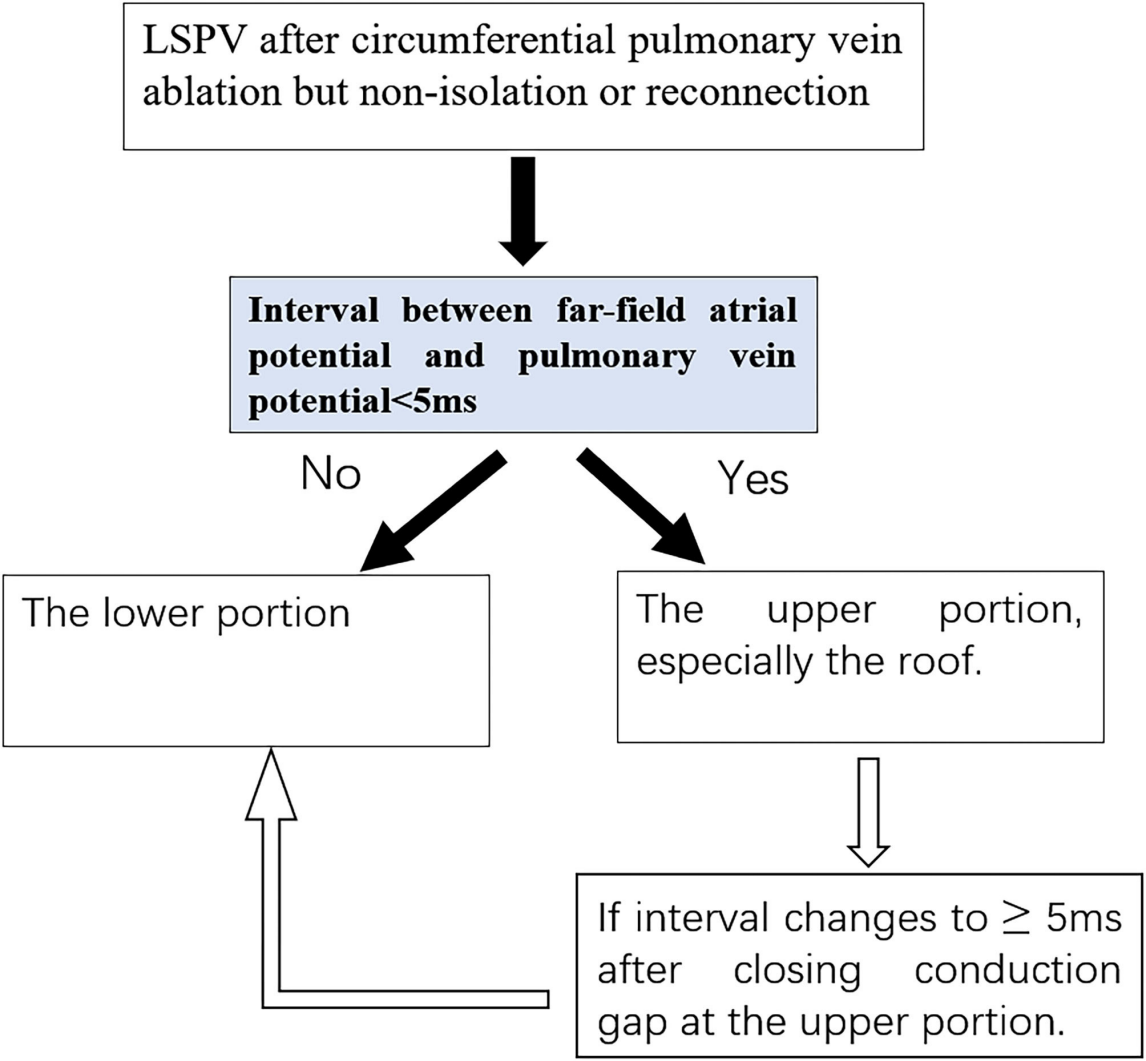
Stepwise diagnostic for locating conduction gap(s).

A previous study reported that conduction over a single gap with a longer conduction time was frequently masked by another gap with a shorter conduction time, when there were more than two gaps with different conduction times (11). In our study, we found that the FFP–PVP intervals were all shorter than 5 ms in eight patients with ≥2 CGs. After eliminating the upper portion gaps, the FFP–PVP interval was suddenly prolonged substantially and the residual gaps finally closed at the lower portion. It seems that the upper portion is the dominant conduction area of the LSPV. Thus, based on the above findings, a novel algorithm was developed for locating CGs (Figure 6). According to this algorithm, once the LSPV is not isolated or reconnected after circumferential PV ablation, the FFP–PVP interval should be measured first. If the interval is longer than 5 ms, a CG located superiorly can be excluded. If the interval is shorter than 5 ms, the CG may be located in the upper portion. When PVP with a delay was detected after eliminating the upper CG, the remaining ones located in the lower portion should be considered.

### A Simple Step for Locating the CG

Although experienced physicians can achieve 100% acute PVI with standard mapping techniques, reconnection between the PV and left atrium is not rare in patients during observation on the table, or after discharge from the hospital (4). Previous studies (4, 12) have underlined the importance of permanent PVI in treating AF. Individuals who have received permanent PV isolation are unlikely to experience an arrhythmia recurrence. In addition, most of the atrial triggers were found to originate in the PVs, particularly in the LSPV, which accounted for 47.7% (2). Hence, CG at LSPV should be focused (13–15). Guided by Nav X (11), Rhythmia (16), and the CARTO mapping system (17), several methods have been reported to allow for acquisition of multiple 3D anatomical points along with the local activation time to visually identify of the CG. However, Miyamoto et al. (11) demonstrated that this visual method could sometimes be compromised by low amplitude and/or fractionated electrograms, which requires additional effort to accurately distinguish between atrial and PV electrograms. As Benito et al. (18) and Masuda et al. (16) reported, the voltage map failed to identify gap localization in two-thirds of the cases. Furthermore, Furnkranz et al. (15) used the interval between P-wave onset and the earliest PVP at Lasso to demonstrate that the CG at the LA roof resulted in no delay or minimal delay of PV activation. However, the timing of P-wave onset to the earliest PVP varied between individuals and was greatly affected by intra-atrial conduction. In this study, we found that there was a short or no delay of PV activation in the presence of a CG at the superior LSPV. We also provided a simple and intuitive method to determine the CG(s) by comparing the FFP–PVP interval. A separation between FFP and PVP suggested that no CG was likely located superiorly. This method is especially valuable for cryoballoon ablation. The combined use of the interval could facilitate the recognition of the CG location without an additional mapping tool, which could decrease the need for touch-ups, and reduce the number of cryoablation applications.

## Study Limitations

This study has several limitations. First, the sample size in the prospective cohort was relatively small and the findings should be further validated in larger populations with multiple centers. Second, although the new algorithm can accurately distinguish between the upper and the lower portion of the ostium for locating CG, the precise location could not be determined. Furthermore, this new algorithm is only applicable to the LSPV, which tends to have a CG. The applicability in other PVs should be further validated.

## Conclusions

The interval between FFP and PVP is a novel and accurate index for predicting the location of a CG in LSPV, and an interval ≥5 ms could exclude a CG in the upper portion of the ostium in a majority of patients.

## Data Availability Statement

The raw data supporting the conclusions of this article will be made available by the authors, without undue reservation.

## Ethics Statement

The studies involving human participants were reviewed and approved by Institutional Review Board of Fuwai Hospital, National Center for Cardiovascular Diseases, Chinese Academy of Medical Sciences and Peking Union Medical College. The patients/participants provided their written informed consent to participate in this study.

## Author Contributions

H-yX and X-gG conceived the study. J-dY and Y-qC analyzed the data. Z-jC collected the data. QS critically revised the article. All authors contributed to the article and approved the submitted version.

## Conflict of Interest

The authors declare that the research was conducted in the absence of any commercial or financial relationships that could be construed as a potential conflict of interest.
